# Interpretable Machine Learning Analysis of Factors Associated with Postoperative Hemoglobin Reduction After Total Knee Arthroplasty: A Standardized-Protocol Cohort Study in Non-Transfused Patients

**DOI:** 10.3390/jcm15155862

**Published:** 2026-07-27

**Authors:** Jae Bum Kwon, Seung Jae Yoo, Junhee Lee, Sang Gyu Kwak, Won Kee Choi

**Affiliations:** 1 Department of Orthopaedic Surgery, School of Medicine, Daegu Catholic University, Daegu 42472, Republic of Korea; kwonjb0630@naver.com (J.B.K.); joy01090@gmail.com (S.J.Y.); june03248@gmail.com (J.L.); 2 Department of Medical Statistics, School of Medicine, Daegu Catholic University, Daegu 42472, Republic of Korea; sgkwak@cu.ac.kr

**Keywords:** total knee arthroplasty, hemoglobin reduction, machine learning, SHAP, tranexamic acid

## Abstract

**Background:** Postoperative hemoglobin (Hb) reduction reflects the physiologic extent of perioperative blood loss after total knee arthroplasty (TKA). Whereas previous studies have relied on transfusion as a binary endpoint, transfusion decisions are highly variable across institutions, obscuring the underlying hematologic trajectory. This study aimed to develop and interpret machine learning (ML) models to characterize and quantify the determinants of postoperative Hb reduction in a standardized cohort of non-transfused TKA patients. **Methods:** A retrospective cohort of 866 patients who underwent primary TKA under a standardized operative protocol—with identical cemented posterior-stabilized implants and uniform cementing technique—was analyzed (1 January 2014–31 March 2024). During the study period, a consistent 1 g intra-articular tranexamic acid (TXA) regimen administered through the drain was introduced and applied to a subset of patients, allowing TXA use to be modeled as a binary predictor. Four ML algorithms (Linear Regression, Random Forest, XGBoost, and Stacking Regressor) were trained using preoperative, demographic, and perioperative variables. Fivefold cross-validation assessed model performance, and SHapley Additive exPlanations (SHAP) values were used to identify influential predictors and enhance interpretability. **Results:** Across all ML models, preoperative Hb emerged as the strongest determinant of postoperative Hb reduction, followed by TXA use, body mass index (BMI), and platelet count. Ensemble models captured non-linear and interacting effects more effectively than linear regression. Test-set performance was modest (best R^2^ = 0.330), consistent with the influence of unmeasured physiologic factors such as hidden blood loss, fluid dynamics, and inflammatory responses. Accordingly, the primary value of the framework lies in the exploratory and transparent assessment of determinant importance rather than in individual-level prediction. **Conclusions:** This study provides an interpretable, exploratory ML framework for identifying factors associated with percentage Hb reduction after TKA. Preoperative Hb was the dominant determinant, while TXA use and higher BMI were recurrently associated with smaller predicted percentage reductions. Given the modest test-set performance and the absence of external validation and clinical utility assessment, the models should not be interpreted as tools for individual-level prediction or clinical decision making.

## 1. Introduction

Total knee arthroplasty (TKA) is one of the most frequently performed orthopedic procedures and provides substantial pain relief and functional restoration, particularly among elderly individuals with end-stage knee osteoarthritis [1]. As the global population continues to age, the annual number of TKAs is steadily increasing worldwide [2]. Despite its well-established benefits, TKA is frequently accompanied by substantial perioperative blood loss—including both visible and hidden components—which may lead to postoperative anemia and occasionally necessitate allogeneic transfusion [3]. However, transfusion is not without risks; it has been associated with higher infection rates, delayed wound healing, prolonged hospitalization, and increased healthcare costs [4]. These concerns have driven ongoing efforts toward evidence-based patient blood management strategies aimed at reducing unnecessary transfusions and optimizing perioperative hemoglobin (Hb) levels [5,6].

Previous studies investigating transfusion risk after TKA have primarily relied on binary transfusion outcomes (yes/no), which inherently depend on institutional or surgeon-specific transfusion policies [7,8]. Transfusion thresholds typically range from 7 to 10 g/dL and are influenced by comorbidities, symptoms, and physician discretion [9]. Such variability introduces significant heterogeneity across studies and limits the generalizability of their findings. Moreover, transfusion-based endpoints fail to capture the physiological continuum of perioperative blood loss and recovery, thereby oversimplifying the multifactorial nature of postoperative anemia.

To overcome these limitations, the present study adopted postoperative Hb reduction as a continuous outcome measure, providing a more objective and quantitative representation of surgical blood loss independent of institutional transfusion thresholds. Patients who received perioperative transfusions were deliberately excluded because transfusion artificially alters Hb levels and obscures the natural physiologic trajectory of Hb change. By restricting the analysis to non-transfused patients, this study aims to delineate intrinsic patient- and surgery-related factors that contribute to Hb reduction, enabling a more accurate understanding of perioperative anemia dynamics.

Recent advances in machine learning (ML) offer new opportunities to model complex non-linear interactions among perioperative variables and enhance predictive accuracy beyond that achievable with conventional regression techniques [10,11]. Furthermore, the application of interpretable ML methods—such as SHapley Additive exPlanations (SHAP)—allows for transparent assessment of the relative importance and direction of associations among candidate predictors [12].

Accordingly, the purpose of this study was to develop interpretable ML models to characterize the magnitude and determinants of postoperative Hb reduction in non-transfused TKA patients. By focusing on a physiologic, transfusion-independent outcome within a standardized surgical setting, this study aimed to provide an exploratory framework for evaluating determinant importance rather than a validated individual-level prediction tool.

## 2. Materials and Methods

### 2.1. Study Subjects

This study included patients who underwent TKA performed by two surgeons at a single center. To accurately evaluate postoperative Hb reduction, patients who received intraoperative or postoperative blood transfusions were excluded from the analysis. This exclusion was necessary because transfusion directly alters the observed postoperative Hb trajectory. However, it restricted the target population to non-transfused patients and excluded many patients with severe preoperative anemia, greater perioperative blood loss, or higher clinical risk. In cases where bilateral TKA was performed during a single hospitalization, only the first surgical procedure was included in the dataset.

### 2.2. Surgical Technique

All procedures were performed under a standardized surgical protocol by two orthopedic surgeons. A pneumatic tourniquet was applied in all patients during surgery to minimize intraoperative bleeding. A cemented posterior-stabilized TKA was performed in all cases through a midline skin incision and medial parapatellar arthrotomy. Following implantation, a closed-suction drain was routinely inserted into the joint. On 1 March 2016, the institution formally introduced a uniform tranexamic acid (TXA) protocol consisting of 1 g (10 mL) administered intra-articularly through the drain, followed by 4 h of drain clamping; TXA was selectively administered thereafter. No patient received TXA before protocol introduction, and the first recorded administration in the analytic cohort occurred on 9 May 2016. After unclamping, the drain was maintained under gentle suction until removal, typically within 24–48 h postoperatively.

### 2.3. Data Collection

The data used in the analysis were collected using the electronic medical record system and the clinical data warehouse (CDW) system of our institution. The collection period was from 1 January 2014 to 31 March 2024, and data were collected from patients who underwent TKA at our institution. If a patient underwent TKA on both sides, only the first surgery was included, and the second was excluded. Age (years), preoperative Hb, sex, body mass index (BMI, kg/m^2^), preoperative platelet count (10^3^/μL), use of anticoagulants, preoperative American Society of Anesthesiologists (ASA) class [13], use of TXA, and anesthesia method (general, regional) were collected. These nine variables constituted the candidate feature set for the ML analyses. Preoperative Hb was modeled as a continuous variable; therefore, a separate binary classification of baseline anemia was not included. Use of anticoagulants was considered present when patients were taking aspirin, clopidogrel, apixaban, heparin, edoxaban, warfarin, dabigatran, or rivaroxaban. For Hb, data were collected before surgery, immediately after surgery, and on postoperative days (POD) 1, 3, 5, and 7. The Hb reduction percentage was calculated as [(preoperative Hb − minimum postoperative Hb)/preoperative Hb] × 100. The percentage Hb reduction served as the dependent variable for all machine learning models. Accordingly, the RMSE values reported for model performance are expressed in percentage points. Absolute Hb change in g/dL was not used as the model outcome and was presented only descriptively for clinical context. To explore the natural changes in Hb levels, subjects who received blood transfusions were excluded from the data. All included predictor and outcome variables had complete data for the entire cohort, with no missing values requiring imputation or case exclusion. This study was approved by the Institutional Review Board of our institution (IRB number 2024-11-003). The requirement for consent was waived because this was a retrospective medical record-based study. All data were fully anonymized before analysis, and no identifiable patient information was accessed.

### 2.4. Statistical Analyses

All statistical analyses were performed using Python 3.7.1 (Python Software Foundation, Wilmington, DE, USA, http://python.org). All values were presented as mean ± standard deviation (SD) for continuous variables and frequency (percent) for categorical variables. Hb levels were presented as mean, SD, minimum, maximum, and median for preoperative, immediately postoperative, and POD 1, 3, 5, and 7 measurements, as well as the minimum value from the immediate to seventh POD and the reduction percentage. A frequency analysis was performed on the POD with the lowest Hb level, and frequencies and percentages were reported.

To evaluate the predictive performance of each model, we employed cross-validation (CV) with fivefold partitioning for training, validation, and testing datasets. Model development used 70% of the cohort for training and internal validation, with the remaining 30% reserved as an independent test set. The root mean squared error (RMSE) and R^2^ scores were used as the primary metrics to assess model accuracy and goodness-of-fit. The CV RMSE (mean ± SD) was calculated for each dataset, and the mean CV R^2^ (mean ± SD) was computed across the respective datasets for each model. Default hyperparameters were used for Linear Regression (LR) and Random Forest (RF), whereas XGBoost (XGB) and the Stacking Regressor were optimized using grid search within the training folds to avoid information leakage.

The performance of the LR, RF, XGB, and Stacking Regressor models was compared in terms of these metrics. A total of 511 candidate feature sets were generated by enumerating all non-empty combinations of the nine available predictors. Each feature set was evaluated separately for each machine learning algorithm using fivefold CV within the development set. For each algorithm, candidate feature sets were ranked solely according to the mean validation-fold R^2^, and the five highest-ranked feature sets were retained. The independent test set was not used for feature-set selection, model ranking, or hyperparameter tuning and was evaluated only after the final model specification had been determined.

Feature importance and the directional effects of predictors were evaluated using SHAP values. For the SHAP analysis, the Rank-1 feature set for each algorithm, as determined exclusively within the development set, was used. SHAP values were computed to assess each predictor’s contribution to the model predictions. For each model, the mean absolute SHAP value was calculated, and the direction of association for each feature was derived from the SHAP summary plot. A positive direction (↑) indicates that higher values of the predictor are associated with an increased postoperative Hb reduction rate, whereas a negative direction (↓) indicates an inverse relationship.

The feature importance values for the Stacking Regressor were computed by averaging the feature importances from the base models (RF and XGB), providing a balanced measure of feature significance. To interpret the predictions of the Stacking Regressor, SHAP values were computed by first calculating SHAP values separately for RF and XGB and then averaging them to obtain a combined measure of feature importance. All codes used for preprocessing and model training will be made publicly available upon reasonable request or upon acceptance to ensure reproducibility. Because the standardized TXA protocol was implemented during the study period, post hoc temporal sensitivity analyses were conducted to assess potential confounding by secular changes in clinical practice. The percentage Hb reduction remained the dependent variable. Multivariable LR models with heteroscedasticity-consistent type 3 (HC3) robust standard errors were fitted by adjusting for calendar year as either a continuous variable or categorical year fixed effects, together with sex, age, BMI, ASA class, preoperative Hb, platelet count, anticoagulant use, and anesthesia type. The analysis was also repeated after restricting the cohort to operations performed on or after 1 March 2016, when both TXA-treated and untreated patients were represented.

As an additional post hoc sensitivity analysis, the four previously identified Rank-1 pipelines were evaluated using a common data split. The dataset was divided into a development set (70%; *n* = 606) and an independent test set (30%; *n* = 260) using a fixed random seed. The same split was applied to all four pipelines, and the independent test set was not used for model selection.

Within the development set, nested cross-validation was performed using five outer folds and fivefold inner cross-validation. In each outer training fold, the four fixed Rank-1 pipelines were compared according to their mean inner-fold R^2^. The pipeline with the highest mean inner-fold R^2^ was fitted using the complete outer training fold and evaluated in the corresponding outer validation fold. Predictions from the five outer validation folds were pooled to estimate the RMSE, R^2^, calibration intercept, and calibration slope. This analysis compared the four fixed Rank-1 pipelines identified in the primary analysis and did not repeat the exhaustive evaluation of all 511 feature combinations within each outer fold.

Calibration was assessed using the regression equation *observed outcome = intercept + slope × predicted outcome*. An intercept of 0 and a slope of 1 indicate ideal calibration. Percentile-based 95% confidence intervals for the performance and calibration metrics were calculated using 2000 non-parametric bootstrap resamples of the paired observed and predicted values. Decision-curve analysis was not performed because no prespecified or clinically validated threshold for percentage Hb reduction linked to a specific clinical intervention was available.

Generative AI (ChatGPT, GPT-5.5; OpenAI, San Francisco, CA, USA) was used only for language editing and clarity improvement. The tool assisted in grammar pol-ishing and stylistic refinement of the manuscript text. No AI tools were used for data analysis, interpretation of results, or scientific decision making. All scientific content, study design, and conclusions were developed entirely by the authors, who take full responsibility for the manuscript.

## 3. Results

### 3.1. Patient Characteristics

A total of 866 patients were included in the study (Table 1). The mean age was 75.00 years (SD ± 7.76). Females constituted the majority, with 688 patients (79.4%), while males accounted for 178 patients (20.6%). The mean BMI was 26.2 kg/m^2^ (SD ± 3.69). The mean preoperative platelet count was 275.93 × 10^3^/μL (SD ± 96.32). Among the patients, 204 (23.6%) were on anticoagulants preoperatively, and 662 (76.4%) were not. The ASA physical status classification revealed that 214 patients (24.7%) were categorized as ASA 1, 596 (68.8%) as ASA 2, and 56 (6.5%) as ASA 3. TXA was used in 222 patients (25.6%), while 644 patients (74.4%) did not receive it. Most patients underwent regional anesthesia (796 patients, 91.9%), whereas general anesthesia was used in 70 patients (8.1%).

### 3.2. Perioperative Hb Changes

**Figure 1:**
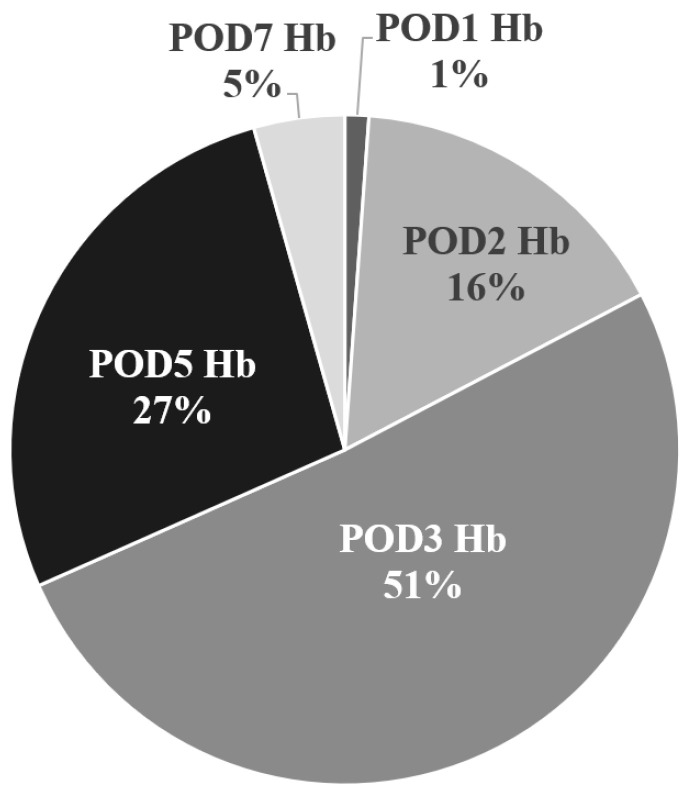
Distribution of the postoperative day on which the minimum hemoglobin value was recorded. Each segment is directly labeled with the corresponding postoperative day and percentage. A high-contrast, color-blind-friendly palette was used, and color is provided only as a visual aid. Hb, hemoglobin; POD, postoperative day.

The mean preoperative Hb level was 13.05 g/dL (SD ± 1.23), which decreased progressively postoperatively (Table 2). Immediately after surgery, the mean Hb level dropped to 11.09 g/dL (SD ± 1.14). On POD 1, 2, and 3, the mean Hb levels were 10.71 g/dL (SD ± 1.16), 9.40 g/dL (SD ± 0.95), and 8.99 g/dL (SD ± 0.97), respectively. Recovery in Hb levels began from POD 5, with a mean value of 9.33 g/dL (SD ± 1.02), and further increased to 9.76 g/dL (SD ± 1.02) on POD 7. The minimum postoperative Hb was 8.80 g/dL (SD ± 0.93). The overall percentage reduction in Hb was 32.22% (SD ± 7.11). This percentage reduction was used as the dependent variable in all machine learning analyses. Figure 1 presents the distribution of the day on which the lowest Hb was recorded. The lowest Hb measurements most frequently occurred on POD 3 (51%), followed by POD 5 (27%), POD 2 (16%), POD 7 (5%), and POD 1 (1%). This distribution emphasizes the concentration of nadir measurements around POD 3, reflecting its clinical relevance as a critical period for monitoring postoperative Hb levels [14].

### 3.3. Performance of ML Models

To identify the best-performing datasets, we analyzed the top five performance datasets for each model based on CV metrics. These results are presented in Supplementary Tables S1–S4 for each model. Each table displays the top five ranked datasets with their respective feature combinations and performance metrics, including CV RMSE, CV R^2^, and final test performance.

For LR (Supplementary Table S1), the top-performing dataset (Rank 1) included Sex, BMI, Preoperative Hb, TXA, and Anesthesia Method, achieving a CV RMSE of 6.034 ± 0.129 and a CV R^2^ of 0.325 ± 0.024. For RF (Supplementary Table S2), the top-performing dataset (Rank 1) included BMI, Preoperative Hb, TXA, and Anesthesia Method, with a CV RMSE of 5.451 ± 0.085 and a CV R^2^ of 0.449 ± 0.017. For XGB (Supplementary Table S3), the best-performing dataset (Rank 1) consisted of Sex, Age, BMI, Preoperative Hb, Preoperative Platelet, and TXA, yielding a CV RMSE of 5.167 ± 0.067 and a CV R^2^ of 0.489 ± 0.018. For the Stacking Regressor (Supplementary Table S4), the top dataset (Rank 1) included BMI, ASA, Preoperative Hb, and TXA, achieving a CV RMSE of 5.391 ± 0.044 and a CV R^2^ of 0.444 ± 0.009.

Table 3 summarizes the Rank-1 feature sets from the four algorithms. In the primary analysis, XGB yielded the lowest training-fold CV RMSE, whereas the Stacking Regressor yielded the lowest validation-fold CV RMSE and the highest test-set R^2^ (0.330). The performance differences between the models were mainly driven by the selected features, with preoperative hemoglobin, BMI, and TXA playing significant roles in predicting Hb reduction.

### 3.4. Feature Importance and SHAP Value Analysis

**Figure 2:**
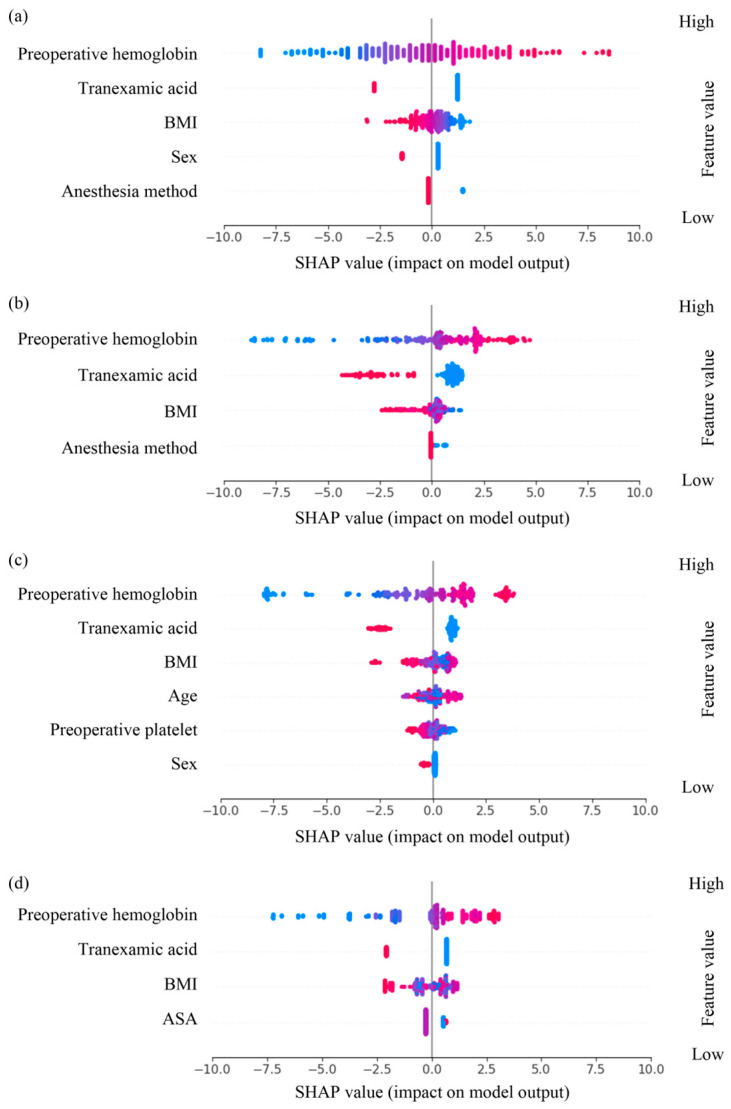
SHAP summary plots for the Rank-1 feature set of each machine learning model: (**a**) linear regression, (**b**) random forest, (**c**) XGBoost, and (**d**) stacking regressor. Each point represents one patient, and its horizontal position indicates the SHAP value for the corresponding predictor. Positive SHAP values indicate an increase in the predicted percentage hemoglobin reduction, whereas negative values indicate a decrease. Point color represents the original feature value, ranging from dark blue for lower values to pink for higher values using a color-blind-friendly continuous scale. For binary predictors, the lower and higher colors correspond to values of 0 and 1, respectively. Preoperative hemoglobin showed the largest SHAP magnitude across the models, whereas higher BMI and TXA use were consistently associated with smaller predicted percentage hemoglobin reductions. SHAP, SHapley Additive exPlanations; BMI, body mass index.

We performed a SHAP analysis to assess the contribution of individual predictors to the model’s predictions. Table 4 summarizes the feature importance, mean absolute SHAP values, and directional effects of the variables across all models. Preoperative Hb emerged as the most important predictor of Hb reduction across all models, with higher preoperative Hb associated with greater reductions in postoperative Hb (↑). BMI and TXA were negatively associated with Hb reduction (↓), suggesting that higher BMI and the administration of TXA may be linked to smaller reductions in Hb levels. Notably, age and sex (male) showed less consistent effects across models. Preoperative platelet count and ASA score also contributed to the models, though their effects were less pronounced. The detailed feature importance and SHAP summary plots for each model are depicted in Figure 2.

### 3.5. Temporal Sensitivity Analysis of TXA Use

TXA was not administered before implementation of the standardized protocol on 1 March 2016. Of the 675 patients who underwent surgery during the post-protocol period, 222 (32.9%) received TXA. The mean percentage Hb reduction decreased from 39.1% in 2014 to 25.5% in 2023; the smaller 2024 cohort (*n* = 15) had a mean reduction of 23.4%. After adjustment for categorical surgery-year fixed effects and clinical covariates, TXA use was associated with a 0.237-percentage-point smaller Hb reduction (95% CI, −0.462 to −0.012; *p* = 0.039). In the analysis restricted to the post-protocol period, the association remained directionally similar but was not statistically significant (β = −0.212 percentage points; 95% CI, −0.438 to 0.015; *p* = 0.067) (Supplementary Table S5).

### 3.6. Post Hoc Validation and Calibration Analyses

In the post hoc nested cross-validation sensitivity analysis, the RF pipeline was selected in all five outer folds. Based on the pooled outer-fold predictions, the RMSE was 5.905 percentage points (95% CI, 5.572–6.238), and the R^2^ was 0.324 (95% CI, 0.266–0.372). The calibration intercept was −4.089 (95% CI, −8.070 to −0.073), and the calibration slope was 1.125 (95% CI, 1.001–1.250).

In the common independent test set, the RF pipeline selected within the development set achieved an RMSE of 5.681 percentage points (95% CI, 5.234–6.137) and an R^2^ of 0.330 (95% CI, 0.236–0.408). Its calibration intercept was −5.288 (95% CI, −11.949 to 0.894), and its calibration slope was 1.147 (95% CI, 0.956–1.347). Test-set performance and calibration results for all four Rank-1 pipelines are presented in Supplementary Table S6, and the corresponding calibration plots are shown in Supplementary Figure S1.

## 4. Discussion

Understanding the determinants of postoperative Hb reduction is essential for optimizing perioperative management in TKA. While previous studies have typically relied on transfusion as the primary endpoint [7,10,11], transfusion decisions are shaped by subjective institutional norms, surgeon preference, and symptom-driven thresholds, making them suboptimal for physiologic modeling [9]. By focusing on postoperative Hb reduction as a continuous outcome, the present study endeavors to characterize the intrinsic hematologic response to TKA more accurately and independently of externally imposed transfusion criteria.

A notable finding in our study was that patients with higher preoperative Hb exhibited a greater percentage reduction in postoperative hemoglobin. This finding refers to a relative change normalized to the preoperative value and should not be interpreted as indicating greater absolute blood loss. Postoperative Hb change reflects the combined effects of red-cell loss, hemodilution, and perioperative fluid shifts. Previous arthroplasty studies have similarly identified preoperative Hb as an important covariate of postoperative Hb change [15,16]. However, because some previous studies evaluated absolute Hb change or estimated blood loss, their findings provide related clinical context rather than directly equivalent evidence for the percentage-based outcome used in the present study. This general relationship is also consistent with hemoglobin-balance methods that estimate red-cell loss using changes in Hb or hematocrit together with estimated blood volume [17,18].

The exclusion of transfused patients introduces an important source of selection bias. Although this exclusion was necessary to examine postoperative Hb trajectories without the direct hematologic effects of transfusion, transfusion is not a random event. Patients with more severe preoperative anemia, greater perioperative blood loss, comorbidities, or symptoms attributable to anemia were more likely to receive transfusion and were therefore disproportionately excluded. Conditioning the analysis on the absence of transfusion may consequently have influenced the observed associations between the candidate predictors and percentage Hb reduction. The findings should therefore be interpreted as applying only to non-transfused TKA patients and cannot be generalized to transfused patients or to the highest-risk population in whom perioperative blood-management decisions are most relevant.

Beyond baseline Hb, TXA use emerged as a recurring predictor across the ML models [19,20,21]. The observed association between TXA use and a smaller percentage Hb reduction is biologically consistent with its antifibrinolytic mechanism, which reduces clot degradation and may attenuate both visible and hidden blood loss [21]. Arthroplasty-specific evidence is consistent with this biologic rationale. An umbrella review of 23 meta-analyses in total hip arthroplasty reported that TXA was associated with reductions in perioperative blood loss, Hb decline, and transfusion requirements without a clear increase in venous thromboembolism or wound complications [22]. However, these findings were derived from hip arthroplasty and predominantly absolute blood-loss and Hb-change outcomes and therefore are not directly equivalent to the percentage Hb reduction evaluated in the present TKA cohort. However, TXA was introduced during the study period and was selectively administered thereafter. Although all treated patients received the same standardized regimen of 1 g intra-articularly through the drain, standardization of dose and route does not eliminate potential confounding by temporal changes in perioperative practice.

After adjustment for surgery year, the estimated association between TXA and percentage Hb reduction was small. Moreover, the confidence interval included the null value when the analysis was restricted to the post-protocol period. These findings indicate that the observed importance of TXA may partly reflect secular changes in perioperative practice, and a causal protective effect cannot be established from the present retrospective data. Calendar-year adjustment was intended to account for secular changes shared by patients treated during the same period; however, it cannot identify or fully control for specific changes in clinical practice. Transfusion thresholds, anesthesia practice, drain management, VTE prophylaxis, mobilization protocols, and perioperative fluid management may have evolved during the 10-year study period. Changes in transfusion practice are particularly relevant because transfused patients were excluded and could therefore have altered the composition of the analytic cohort over time. Although anesthesia type was included as a covariate and the core surgical technique was broadly standardized, detailed longitudinal information on the other practice-related factors was unavailable. Consequently, residual temporal confounding cannot be excluded. Anticoagulant use was represented as a single binary predictor. The specific agent, indication, perioperative interruption and resumption schedule, bridging therapy, and postoperative VTE prophylaxis were not separately modeled. Therefore, the present findings cannot determine the effects of specific anticoagulant-management pathways on percentage Hb reduction.

The association between lower BMI and greater Hb reduction is also supported by physiologic evidence. Patients with lower BMI often have reduced circulating blood volume, lower muscle mass, and diminished microvascular integrity, all of which may increase susceptibility to hemodilution or postoperative inflammatory extravasation [23]. BMI may therefore serve as a surrogate marker for physiologic reserve and vascular robustness. Preoperative platelet count demonstrated a smaller but meaningful contribution, consistent with established pathways of coagulative integrity and thrombin generation [3]. Regional anesthesia was also associated with a modest reduction in Hb decline relative to general anesthesia; this may reflect the neuraxial sympathetic blockade and lower intraoperative blood pressure characteristic of spinal or epidural techniques, which can reduce venous engorgement and operative-site bleeding compared with the hemodynamic and fluid-management profile typical of general anesthesia.

Machine learning models provided distinct advantages over traditional linear approaches. LR assumes proportional, additive, and independent effects among predictors—assumptions rarely satisfied in perioperative physiology, where Hb reduction results from non-linear interactions among inflammatory cascades, endothelial permeability, surgical tissue trauma, microvascular bleeding, and postoperative fluid balance. Ensemble ML algorithms such as RF, XGB, and Stacking are better suited to capturing these high-order interactions and threshold-dependent effects. In this study, these models demonstrated higher and more stable predictive performance across CV.

Given that several potentially influential determinants of postoperative hematologic change—including intraoperative blood loss, fluid administration, capillary leak, and patient-specific inflammatory responses—were not captured, the models demonstrated only modest test-set performance. The best test-set R^2^ of 0.330 indicates that a substantial proportion of individual variability remained unexplained. Accordingly, the present models should be interpreted as an exploratory framework for comparing determinant importance and examining potential non-linear associations, rather than as tools for individual-level prediction or clinical decision making.

Because all 511 candidate feature combinations were evaluated, the possibility of feature-selection bias and model-selection optimism warrants consideration. Several safeguards were implemented: feature-set selection was based on fivefold CV conducted exclusively within the development set, the independent 30% hold-out test set was not used during feature selection or hyperparameter tuning, and grid-search optimization for XGB and the stacking regressor was confined to the training folds to prevent information leakage. Nevertheless, because the same development dataset was used to compare a large number of candidate feature sets, some degree of feature-selection bias and model-selection optimism cannot be completely excluded. The repeated selection of preoperative hemoglobin, TXA use, and BMI among the highest-ranked feature sets across algorithms with different modeling assumptions provides supportive, although not definitive, evidence that these findings are robust rather than chance-driven. The consistency of the SHAP-derived directions with established clinical and physiologic relationships further supports the stability of the observed associations.

In the post hoc common-split validation analyses, the RF pipeline was selected in all five outer folds of the nested cross-validation analysis and showed an R^2^ of 0.330 in the common independent test set. The 95% confidence intervals for its test-set calibration intercept and slope included their respective ideal values of 0 and 1, although the intervals were relatively wide. In contrast, the pooled nested cross-validation predictions showed a modest deviation from ideal calibration. Together with an RMSE of approximately 5.7 percentage points, these findings indicate that uncertainty remains in individual-level predictions.

The design of this study also provides several unique strengths. The complete standardization of implant type, cement type, surgical technique, and TXA protocol eliminates key sources of operative heterogeneity and reduces confounding related to procedural choices—an uncommon advantage in retrospective orthopedic datasets. Additionally, the deliberate exclusion of transfused patients, although reducing applicability to the highest-risk group, enables evaluation of unaltered Hb trajectories, free from the abrupt discontinuities introduced by transfusion. This physiologic purity allows clearer interpretation of how baseline and perioperative factors shape Hb decline.

These determinant-level findings should be interpreted as exploratory rather than prescriptive. Preoperative Hb was the dominant contributor to the predicted percentage Hb reduction; however, percentage reduction does not directly represent the absolute severity of postoperative anemia and should be interpreted together with the postoperative Hb level and the patient’s clinical condition. Although TXA use was recurrently associated with a smaller percentage Hb reduction in the primary ML models, this association was attenuated after accounting for calendar time and does not establish an independent treatment effect. Similarly, the observed concentration of the Hb nadir around POD 3 describes the cohort-level trajectory but does not establish the optimal timing of Hb monitoring for individual patients. Prospective studies incorporating clinical outcomes and utility analyses are needed before these findings can be used to guide perioperative management.

Nevertheless, important limitations warrant consideration. Excluding transfused patients introduced selection bias and restricted the applicability of the findings to a selected, lower-risk population. Because transfusion is associated with baseline anemia, perioperative blood loss, comorbidities, and clinical symptoms, the predictor–outcome relationships observed among non-transfused patients may not apply to patients who undergo transfusion. Accordingly, the present models should not be used to estimate transfusion risk or guide perioperative blood-management decisions in high-risk patients. Future studies should include both transfused and non-transfused patients and apply analytic approaches that account for the timing and hematologic effects of transfusion.

Several potentially important surgical and physiologic variables were not incorporated into the modeling framework. Although a pneumatic tourniquet was used in all patients, exact tourniquet time was not included. Operative time, drain output, estimated blood loss, intraoperative fluid administration, postoperative fluid balance, renal function measures, and perioperative iron therapy were also not included in the candidate feature set. Preoperative Hb was modeled continuously and therefore partially represented baseline hematologic status, but a separate categorical definition of baseline anemia was not evaluated. Furthermore, although all procedures followed the same core surgical protocol and were performed by two surgeons, surgeon identity and inter-surgeon variability were not explicitly modeled. These omissions may have introduced residual confounding and likely contributed to the unexplained individual variability and modest test-set performance. Future prospective, multicenter studies should incorporate these surgical, hemodynamic, and patient-level factors.

Furthermore, although key operative parameters were standardized, other intraoperative variables—such as fluid balance, vasopressor use, or real-time quantification of blood loss—were not captured in this retrospective dataset. Incorporating these physiologic and hemodynamic variables into future models may deepen the mechanistic understanding of postoperative anemia.

The single-center design also limits generalizability; external multicenter and temporal validation across evolving clinical practice patterns will be essential to strengthen robustness. Future work will include multicenter datasets that incorporate variability in surgical technique, perioperative management, and patient characteristics to assess the robustness and clinical applicability of the model.

The post hoc nested cross-validation sensitivity analysis compared only the four Rank-1 pipelines previously identified in the primary analysis and did not repeat the exhaustive evaluation of all 511 feature combinations within each outer fold. Moreover, the common-split analysis used a resampling framework different from that of the primary analysis, and its estimates should therefore not be interpreted as confidence intervals for the point estimates reported in Table 3. Decision-curve analysis was not performed because no prespecified or clinically validated percentage Hb reduction threshold linked to a specific intervention was available. Consequently, the clinical net benefit of the models remains unknown.

Finally, although Hb reduction provides a quantitative and physiologic outcome, it is not itself a clinical endpoint. The present analyses do not establish the clinical utility of the models. Assessment of net benefit would require a clinically actionable threshold for percentage Hb reduction, a corresponding intervention, and explicit consideration of the consequences of false-positive and false-negative decisions. Until such a decision context is prospectively defined, the models should be regarded as exploratory prediction and determinant-analysis tools rather than clinical decision-support systems.

## 5. Conclusions

This study provides an interpretable ML framework for exploring factors associated with percentage Hb reduction among non-transfused patients undergoing TKA within a standardized surgical setting. Preoperative Hb emerged as the dominant determinant, while TXA use and higher BMI were recurrently associated with smaller predicted percentage reductions. However, the modest test-set performance and attenuation of the TXA association after adjustment for calendar time warrant cautious interpretation. The framework should therefore be regarded as an exploratory determinant-analysis approach rather than a validated individual-level prediction tool. External multicenter and temporal validation, further assessment of calibration in independent cohorts, and prospective evaluation of clinical utility will be required before clinical application.

## Figures and Tables

**Table 1:** Patient characteristics and surgical factors.

Variable		Values
Number of Patients		866
Age (years)		75 ± 7.76
Sex	F	688 (79.4)
	M	178 (20.6)
Preoperative Hb (g/dL)		13.05 ± 1.23
BMI (kg/m^2^)		26.2 ± 3.69
Preoperative platelet (10^3^/μL)		275.93 ± 96.32
Anticoagulants	No	662 (76.4)
	Yes	204 (23.6)
ASA	1	214 (24.7)
	2	596 (68.8)
	3	56 (6.5)
Tranexamic acid	No	644 (74.4)
	Yes	222 (25.6)
Anesthesia method	General	70 (8.1)
	Regional	796 (91.9)

All values are presented as mean ± standard deviation for continuous variables and frequency (percent) for categorical variables.

**Table 2:** Preoperative and postoperative hemoglobin levels, including minimum postoperative hemoglobin and hemoglobin reduction percent: descriptive statistics for mean, standard deviation, minimum, maximum, and median.

Variable	Mean	Standard Deviation	Minimum	Maximum	Median
Preop Hb	13.05	1.23	9.40	16.90	13.10
Just after OP Hb	11.09	1.14	7.90	14.60	11.00
POD1 Hb	10.71	1.16	7.60	14.50	10.60
POD2 Hb	9.40	0.95	7.20	12.50	9.30
POD3 Hb	8.99	0.97	7.00	12.30	8.90
POD5 Hb	9.33	1.02	7.10	12.90	9.20
POD7 Hb	9.76	1.02	7.60	13.50	9.70
Minimum postoperative Hb	8.80	0.93	7.00	11.80	8.70
Hb reduction (%)	32.22	7.11	8.04	50.94	32.50

Hemoglobin reduction (%) was calculated as [(preoperative Hb − minimum postoperative Hb)/preoperative Hb] × 100. Percentage hemoglobin reduction was used as the dependent variable in all machine learning analyses. Hb, hemoglobin; OP, operation; POD, postoperative day.

**Table 3:** Comparison of the Rank-1 machine learning models for predicting percentage hemoglobin reduction.

Model (Features)	Train		Validation		Test	
	CV RMSE (Mean ± SD)	CV R^2^ (Mean ± SD)	CV RMSE (Mean ± SD)	CV R^2^ (Mean ± SD)	RMSE	R^2^
Linear regression (Sex, BMI, Preop Hb, TXA, Anesthesia method)	6.034 ± 0.129	0.325 ± 0.024	6.092 ± 0.482	0.304 ± 0.088	5.902	0.173
Random forest regressor (BMI, Preop Hb, TXA, Anesthesia method)	5.451 ± 0.085	0.449 ± 0.017	5.879 ± 0.432	0.352 ± 0.076	5.620	0.250
XGB regressor (Sex, Age, BMI, Preop Hb, Preop platelet, TXA)	5.167 ± 0.067	0.489 ± 0.018	5.866 ± 0.244	0.332 ± 0.048	5.816	0.270
Stacking regressor (BMI, ASA, Preop Hb, TXA)	5.391 ± 0.044	0.444 ± 0.009	5.823 ± 0.149	0.350 ± 0.019	5.573	0.330

For each algorithm, the Rank-1 feature set was selected solely on the basis of the mean validation-fold R^2^ obtained within the development set. The independent test set was not used for feature-set selection or model ranking. RMSE values are expressed in percentage points. CV, cross-validation; Hb, hemoglobin; RMSE, root mean squared error; SD, standard deviation; TXA, Tranexamic acid; R^2^, coefficient of determination.

**Table 4:** Comparative analysis of feature importance, mean absolute SHAP values, and directional effects of predictors on postoperative hemoglobin reduction rate across machine learning models.

Predictor Variable	Feature Importance				Mean (|SHAP Value|)				Direction of Association			
	LR	RF	XGB	Stacking	LR	RF	XGB	Stacking	LR	RF	XGB	Stacking
Age			0.122				0.511				↓	
Sex (Male)	1.725		0.074		0.512		0.152		↓		↓	
BMI	0.216	0.151	0.117	0.173	0.589	0.473	0.613	0.717	↓	↓	↓	↓
Preoperative platelet			0.104				0.367				↓	
Preoperative hemoglobin	2.994	0.669	0.319	0.539	2.737	2.179	2.140	1.713	↑	↑	↑	↑
ASA				0.061				0.359				↑
Tranexamic acid (Yes)	4.012	0.170	0.264	0.226	1.619	1.449	1.286	1.058	↓	↓	↓	↓
Anesthesia method (Regional)	1.658	0.008			0.293	0.085			↓	↓		

The arrows (↑/↓) indicate the direction of each predictor’s effect on postoperative hemoglobin reduction rate as interpreted from the SHAP summary plots: ↑ indicates that higher values of the variable are associated with greater postoperative hemoglobin reduction (higher reduction rate), while ↓ indicates an association with smaller reduction. The feature importance values and mean SHAP values for the stacking regressor were computed by averaging those from the base models (random forest and XGBoost). LR, linear regression; RF, random forest; XGB, XGBoost.

## Data Availability

The data presented in this study are available on reasonable request from the corresponding author. The data are not publicly available due to privacy restrictions.

## Supplementary Materials

The following supporting information can be downloaded at: https://www.mdpi.com/article/10.3390/jcm15155862/s1, Table S1: Performance analysis results of a linear regression model predicting the rate of hemoglobin reduction using patient characteristics and surgical factors; Top 5 performance datasets among 511 datasets; Table S2: Performance analysis results of a random forest regressor model predicting the rate of hemoglobin reduction using patient characteristics and surgical factors; Top 5 performance datasets among 511 datasets; Table S3: Performance analysis results of a XGB regressor model predicting the rate of hemoglobin reduction using patient characteristics and surgical factors; Top 5 performance datasets among 511 datasets; Table S4: Performance analysis results of a Stacking regressor model predicting the rate of hemoglobin reduction using patient characteristics and surgical factors; Top 5 performance datasets among 511 datasets; Table S5: Annual temporal distribution and sensitivity analyses of the association between tranexamic acid use and postoperative percentage hemoglobin reduction; Table S6: Post hoc common-split validation, calibration, and nested cross-validation sensitivity analyses of the four Rank-1 pipelines; Figure S1: Calibration plots of the four Rank-1 pipelines in the common independent test set.

## Abbreviations

The following abbreviations are used in this manuscript:

ASA	American Society of Anesthesiologists
BMI	Body mass index
CI	Confidence interval
CV	Cross-validation
Hb	Hemoglobin
LR	Linear regression
ML	Machine learning
OP	Operation
POD	Postoperative day
RF	Random forest
RMSE	Root mean squared error
SD	Standard deviation
SHAP	Shapley Additive explanations
TKA	Total knee arthroplasty
TXA	Tranexamic acid
XGB	Xgboost
