# Supplementary material for: Interpretable Machine Learning Analysis of Factors Associated with Postoperative Hemoglobin Reduction After Total Knee Arthroplasty: A Standardized-Protocol Cohort Study in Non-Transfused Patients
Source: J Clin Med. 2026 Jul 27;15(15):5862. doi: 10.3390/jcm15155862 (PMC13466657; doi:10.3390/jcm15155862)
Supplement: Supplementary file 1 [file jcm-15-05862-s001.zip › jcm-4443322-supplementary.pdf]

Supplementary Table S1. Performance analysis results of a linear regression model predicting the rate of hemoglobin reduction using patient characteristics and surgical factors; Top 5 performance datasets among 511 datasets.

Rank	Dataset ID	Features	Train		Validation		Test	
			CV RMSE (Mean±SD)	CV R^2 (Mean±SD)	CV RMSE (Mean±SD)	CV R^2 (Mean±SD)	RMSE	R^2
1	306	Sex, BMI, Preop Hb, Tranexamic acid, Anesthesia method	6.034±0.129	0.325±0.024	6.092±0.482	0.304±0.088	5.902	0.173
2	430	Sex, BMI, Preop Hb, Anticoagulants, Tranexamic acid, Anesthesia method	6.031±0.127	0.326±0.024	6.097±0.482	0.303±0.089	5.915	0.169
3	158	Sex, BMI, Preop Hb, Tranexamic acid	6.050±0.129	0.322±0.024	6.102±0.488	0.301±0.089	5.888	0.177
4	304	Sex, BMI, Preop Hb, Anticoagulants, Tranexamic acid	6.047±0.128	0.322±0.024	6.107±0.484	0.300±0.090	5.900	0.174
5	374	BMI, Preop Hb, Anticoagulants, Tranexamic acid, Anesthesia method	6.034±0.113	0.325±0.022	6.108±0.436	0.300±0.084	5.989	0.149

CV: cross validation; Hb: hemoglobin; RMSE: root mean squared error; SD: standard deviation.

Supplementary Table S2. Performance analysis results of a random forest regressor model predicting the rate of hemoglobin reduction using patient characteristics and surgical factors; Top 5 performance datasets among 511 datasets.

Rank	Dataset ID	Features	Train		Validation		Test	
			CV RMSE (Mean±SD)	CV R^2 (Mean±SD)	CV RMSE (Mean±SD)	CV R^2 (Mean±SD)	RMSE	R^2
1	236	BMI, Preop Hb, Tranexamic acid, Anesthesia method	5.451±0.085	0.449±0.017	5.879±0.432	0.352±0.076	5.620	0.250
2	234	BMI, Preop Hb, Anticoagulants, Tranexamic acid	5.401±0.098	0.459±0.020	5.881±0.467	0.351±0.084	5.603	0.255
3	102	BMI, Preop Hb, Tranexamic acid	5.440±0.088	0.452±0.017	5.893±0.423	0.349±0.071	5.598	0.256
4	158	Sex, BMI, Preop Hb, Tranexamic acid	5.426±0.094	0.454±0.017	5.891±0.434	0.349±0.079	5.595	0.257
5	430	Sex, BMI, Preop Hb, Anticoagulants, Tranexamic acid, Anesthesia method	5.441±0.097	0.451±0.018	5.909±0.456	0.345±0.083	5.606	0.254

CV: cross validation; RMSE: root mean squared error.

Supplementary Table S3. Performance analysis results of a XGB regressor model predicting the rate of hemoglobin reduction using patient characteristics and surgical factors; Top 5 performance datasets among 511 datasets.

Rank	Dataset ID	Features	Train		Validation		Test	
			CV RMSE (Mean±SD)	CV R^2 (Mean±SD)	CV RMSE (Mean±SD)	CV R^2 (Mean±SD)	RMSE	R^2
1	393	Sex, Age, BMI, Preop Hb, Preoperative platelet, Tranexamic acid	5.167±0.067	0.489±0.018	5.866±0.244	0.332±0.048	5.816	0.270
2	443	Age, BMI, ASA, Preop Hb, Tranexamic acid, Anesthesia method	5.363±0.101	0.442±0.015	5.820±0.503	0.331±0.030	5.698	0.325
3	397	Sex, Age, BMI, Preop Hb, Tranexamic acid, Anesthesia method	5.368±0.098	0.441±0.012	5.827±0.490	0.329±0.028	5.675	0.330
4	511	Sex, Age, BMI, ASA, Preop Hb, Preoperative platelet, Anticoagulants, Tranexamic acid, Anesthesia method	5.176±0.072	0.488±0.018	5.883±0.27	0.328±0.045	5.842	0.264
5	395	Sex, Age, BMI, Preop Hb, Anticoagulants, Tranexamic acid	5.364±0.101	0.442±0.012	5.835±0.510	0.328±0.027	5.704	0.323

CV: cross validation; RMSE: root mean squared error.

Supplementary Table S4. Performance analysis results of a Stacking regressor model predicting the rate of hemoglobin reduction using patient characteristics and surgical factors; Top 5 performance datasets among 511 datasets.

Rank	Dataset ID	Features	Train		Validation		Test	
			CV RMSE (Mean±SD)	CV R^2 (Mean±SD)	CV RMSE (Mean±SD)	CV R^2 (Mean±SD)	RMSE	R^2
1	223	BMI, ASA, Preop Hb, Tranexamic acid	5.391±0.044	0.444±0.009	5.823±0.149	0.350±0.019	5.573	0.330
2	462	BMI, ASA, Preop Hb, Anticoagulants, Tranexamic acid, Anesthesia method	5.393±0.057	0.443±0.010	5.837±0.130	0.347±0.017	5.549	0.336
3	364	BMI, ASA, Preop Hb, Anticoagulants, Tranexamic acid	5.409±0.048	0.440±0.010	5.843±0.137	0.346±0.017	5.582	0.328
4	366	BMI, ASA, Preop Hb, Tranexamic acid, Anesthesia method	5.449±0.058	0.431±0.010	5.850±0.137	0.344±0.015	5.588	0.326
5	393	Sex, Age, BMI, Preop Hb, Preoperative platelet, Tranexamic acid	4.938±0.097	0.534±0.024	5.855±0.101	0.344±0.036	5.852	0.261

CV: cross validation; RMSE: root mean squared error.

Supplementary Table S5. Annual temporal distribution and sensitivity analyses of the association between tranexamic acid use and postoperative percentage hemoglobin reduction.

Panel A. Annual distribution of TXA use and percentage hemoglobin reduction

Surgery year	N	TXA use, n (%)	Percentage Hb reduction, mean ± SD
2014	83	0 (0.0)	39.1 ± 5.7
2015	98	0 (0.0)	36.7 ± 6.9
2016	72	14 (19.4)	35.7 ± 6.8
2017	72	26 (36.1)	32.6 ± 6.5
2018	84	21 (25.0)	33.4 ± 5.6
2019	84	25 (29.8)	32.2 ± 5.3
2020	83	26 (31.3)	30.5 ± 5.3
2021	89	26 (29.2)	30.8 ± 5.0
2022	94	26 (27.7)	28.5 ± 5.5
2023	92	52 (56.5)	25.5 ± 6.4
2024	15	6 (40.0)	23.4 ± 4.1

Panel B. Temporal sensitivity analyses of the association between TXA use and percentage hemoglobin reduction

Model	N	β	Robust SE	95% CI	p-value
Linear-time-adjusted full cohort	866	−0.473	0.164	−0.794 to −0.152	0.004
Year-fixed-effect full cohort	866	−0.237	0.115	−0.462 to −0.012	0.039
Post-protocol-period restricted	675	−0.212	0.116	−0.438 to 0.015	0.067

The standardized TXA protocol was introduced on 1 March 2016; the post-protocol-period analysis was restricted to operations performed on or after this date. In Panel B, β represents the adjusted mean difference in percentage hemoglobin reduction for TXA use versus no TXA use; a negative value indicates a smaller percentage reduction with TXA. All models were adjusted for sex, age, BMI, ASA class, preoperative Hb, platelet count, anticoagulant use, and anesthesia type. The linear-time model included centered surgery year as a continuous variable, whereas the year-fixed-effect models included surgery year as a categorical variable. CI, confidence interval; Hb, hemoglobin; SD, standard deviation; SE, standard error; TXA, tranexamic acid.

Supplementary Table S6. Post hoc common-split validation, calibration, and nested cross-validation sensitivity analyses of the four Rank-1 pipelines.

Panel A. Performance and calibration in the common independent test set

Model	RMSE (95% CI)	R^2 (95% CI)	Calibration intercept (95% CI)	Calibration slope (95% CI)
Linear regression	5.852 (5.375–6.332)	0.289 (0.187–0.369)	−0.866 (−6.274 to 5.254)	1.017 (0.831–1.192)
Random forest regressor	5.681 (5.234–6.137)	0.330 (0.236–0.408)	−5.288 (−11.949 to 0.894)	1.147 (0.956–1.347)
XGB regressor	5.653 (5.193–6.109)	0.336 (0.254–0.404)	−9.981 (−17.414 to −2.839)	1.295 (1.077–1.522)
Stacking regressor	6.493 (5.954–7.029)	0.124 (0.060–0.175)	−16.745 (−32.211 to −1.453)	1.510 (1.035–1.993)

Panel B. Model selection within the development set in the post hoc common-split analysis

Model	CV R^2, mean (SD)	Rank
Random forest regressor	0.318 (0.065)	1
XGB regressor	0.297 (0.056)	2
Linear regression	0.265 (0.063)	3
Stacking regressor	0.159 (0.027)	4

Panel C. Nested cross-validation sensitivity analysis (n = 606)

Analysis	RMSE (95% CI)	R^2 (95% CI)	Calibration intercept (95% CI)	Calibration slope (95% CI)
Five outer folds with five-fold inner cross-validation	5.905 (5.572–6.238)	0.324 (0.266–0.372)	−4.089 (−8.070 to −0.073)	1.125 (1.001–1.250)

Panel D. Pipeline selection frequency across the five outer folds

Pipeline	Outer folds selected, n	Selection frequency, %
Linear regression	0	0
Random forest regressor	5	100
XGB regressor	0	0
Stacking regressor	0	0

All four pipelines were evaluated using the same post hoc split (development set, n = 606; independent test set, n = 260), with model selection based solely on development-set CV. Panel B results are distinct from those in Table 3. Nested CV compared the four fixed Rank-1 pipelines without repeating the search across all 511 feature combinations. The 95% CIs were calculated using 2,000 paired bootstrap resamples. Calibration was estimated as observed outcome = intercept + slope × predicted outcome (ideal values: intercept = 0 and slope = 1). RMSE is expressed in percentage points. Because a different resampling framework was used, these CIs do not apply to the estimates in Table 3. CI, confidence interval; CV, cross-validation; RMSE, root mean squared error; SD, standard deviation; XGB, extreme gradient boosting.

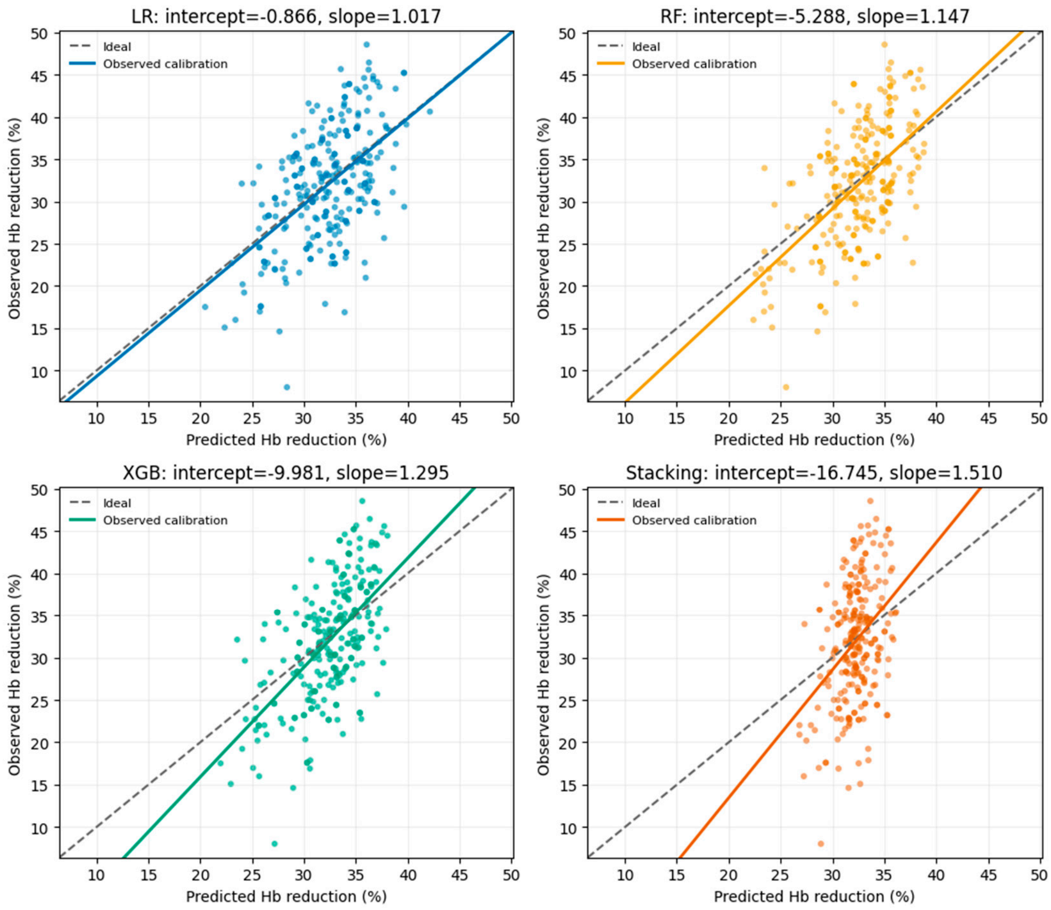

Supplementary Figure S1. Calibration plots of the four Rank-1 pipelines in the common independent test set.
